# Chemical-Genetic Interactions of *Bacopa monnieri* Constituents in Cells Deficient for the DNA Repair Endonuclease *RAD1* Appear Linked to Vacuolar Disruption

**DOI:** 10.3390/molecules26051207

**Published:** 2021-02-24

**Authors:** Chananya Huangteerakul, Hsu Mon Aung, Thitipa Thosapornvichai, Marisa Duangkaew, Amornrat Naranuntarat Jensen, Suchada Sukrong, Kornkanok Ingkaninan, Laran T. Jensen

**Affiliations:** 1 Department of Biochemistry, Faculty of Science, Mahidol University, Bangkok 10400, Thailand; c.huangteerakul@gmail.com (C.H.); drhsumonaung87@gmail.com (H.M.A.); pa_lim_piim@hotmail.com (T.T.); 2 Toxicology Graduate Program, Faculty of Science, Mahidol University, Bangkok 10400, Thailand; who-miss@hotmail.com; 3 Department of Pathobiology, Faculty of Science, Mahidol University, Bangkok 10400, Thailand; amornrat.nar@mahidol.edu; 4 Center of Excellence on Environmental Health and Toxicology (EHT), Bangkok 10400, Thailand; 5 Research Unit of DNA Barcoding of Thai Medicinal Plants, Department of Pharmacognosy and Pharmaceutical Botany, Faculty of Pharmaceutical Sciences, Chulalongkorn University, Bangkok 10400, Thailand; suchada.su@chula.ac.th; 6 Department of Pharmaceutical Chemistry and Pharmacognosy, Faculty of Pharmaceutical Sciences and Center of Excellence for Innovation in Chemistry, Naresuan University, Phitsanulok 65000, Thailand; k_ingkaninan@yahoo.com

**Keywords:** *Bacopa monnieri*, colorectal cancer, synthetic lethality, chemical genetics, yeast, bacopasaponin C

## Abstract

Colorectal cancer is a common cancer worldwide and reduced expression of the DNA repair endonuclease XPF (xeroderma pigmentosum complementation group F) is associated with colorectal cancer. *Bacopa monnieri* extracts were previously found to exhibit chemical-genetic synthetic lethal effects in a *Saccharomyces cerevisiae* model of colorectal cancer lacking Rad1p, a structural and functional homologue of human XPF. However, the mechanisms for *B. monnieri* extracts to limit proliferation and promote an apoptosis-like event in *RAD1* deleted yeast was not elucidated. Our current analysis has revealed that *B. monnieri* extracts have the capacity to promote mutations in *rad1*∆ cells. In addition, the effects of *B. monnieri* extracts on *rad1*∆ yeast is linked to disruption of the vacuole, similar to the mammalian lysosome. The absence of *RAD1* in yeast sensitizes cells to the effects of vacuole disruption and the release of proteases. The combined effect of increased DNA mutations and release of vacuolar contents appears to induce an apoptosis-like event that is dependent on the meta-caspase Yca1p. The toxicity of *B. monnieri* extracts is linked to sterol content, suggesting saponins may be involved in limiting the proliferation of yeast cells. Analysis of major constituents from *B. monnieri* identified a chemical-genetic interaction between bacopasaponin C and *rad1*∆ yeast. Bacopasaponin C may have potential as a drug candidate or serve as a model for the development of analogs for the treatment of colorectal cancer.

## 1. Introduction

The incidence of colorectal cancer is increasing worldwide, due in part to changes in diet and lifestyle [1,2,3]. Surgery, radiotherapy, chemotherapy, or a combination of these strategies are utilized in the treatment of colorectal cancer [4]. While surgery is the most common treatment, advanced disease limits its effectiveness. In many cases, the use of chemotherapy is needed to prevent cancer recurrence [4,5].

Commonly used chemotherapeutic drugs, such as 5-fluorouracil, capecitabine (metabolized to 5-fluorouracil), and oxaliplatin [5,6,7], do not specifically target cancer cells [7,8]. Resulting side effects from generalized toxicity are often difficult for patients to tolerate [5,9,10,11,12]. Targeted therapies that exploit differences between cancer and normal cells are thus desirable [13,14].

A difference between normal cells and a subset of colorectal cancers is reduced expression of *ERCC4* (excision repair cross-complementation group 4), also called DNA repair endonuclease *XPF* [15,16]. XPF functions in nucleotide excision repair (NER) and double-strand break (DSB) repair pathways [15,17]. Previously, we utilized a *Saccharomyces cerevisiae* model of colorectal cancer lacking Rad1p, a structural and functional homologue of human XPF [15,17,18]. Screening medicinal plant extracts for chemical-genetic interactions in *Saccharomyces cerevisiae* deleted for *RAD1* identified *Bacopa monnieri (L.) Wettst* as a potential source of drug candidates. Exposure of *rad1*∆ yeast, but not wild type cells, to *B. monnieri extracts* resulted in nuclear fragmentation suggesting the promotion of an apoptosis-like event [19].

*B. monnieri* is a widely utilized medicinal plant from the Indian Ayurvedic tradition [20]. Preparations containing *B. monnieri* are used to promote cognitive performance and improve memory [21,22]. In addition, *B. monnieri* extracts have been found to exhibit anti-tumor activity [23,24]. The best characterized bioactive compounds from *B. monnieri* are saponins, referred to as bacosides, bacopasides, and bacopasaponins [25,26,27]. The saponin fraction from *B. monnieri* extracts contains bacopaside I, bacoside A_3_, bacopaside II, bacopasaponin X, and bacopasaponin C [28]. We speculated that one or more of these compounds was responsible for promoting an apoptosis-like event and specifically limiting growth in yeast deleted for *RAD1* [19]. These bioactive compounds from *B. monnieri* may have potential as drug candidates for the treatment of *ERCC4* deficient colorectal cancer.

In this study, we investigate the molecular mechanisms that promote the apoptosis-like event following exposure to *B. monnieri* extract, using the yeast model for *ERCC4* deficient colorectal cancer. Our analysis indicates that *B. monnieri* extracts increase mutation frequency but not chromosomal instability in yeast deleted for *RAD1*. Loss of integrity of the yeast vacuole, similar to the mammalian lysosome, was observed. The toxicity of *B. monnieri* extracts was dependent on sterol content, suggesting a role for bacosides in limiting the proliferation of yeast cells. Analysis of major constituents from *B. monnieri* indicates that bacopasaponin C is the bioactive compound responsible for preferentially limiting the growth of *RAD1* deleted yeast. We propose a model in which bacopasaponin C, and perhaps other bioactive compounds from *B. monnieri,* promotes disruption of the vacuole leading to induction of an apoptosis-like event facilitated by leakage of vacuolar components.

## 2. Results

### 2.1. Mutation Frequency But Not Chromosome Instability Is Enhanced Following Exposure to B. monnieri Extracts

**Figure 1:**
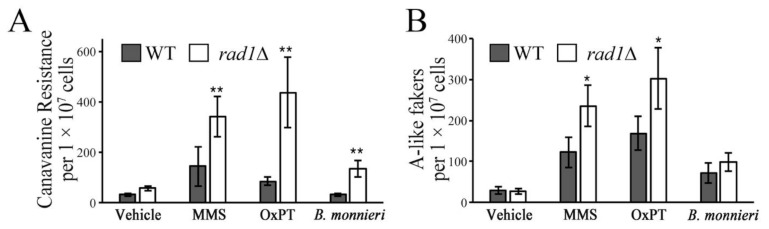
Extracts from *B. monnieri* increase mutation frequency in *rad1*∆ yeast. Strains WT (BY4742) and *rad1*∆ (12806) were grown in synthetic medium lacking arginine with 2% glucose in the presence of 2% DMSO and 0.2% Tween 80 (vehicle control) or exposed 325 µg/mL *B. monnieri* extract, 300 µM MMS, or 2.5 mM oxaliplatin for 16 h. (**A**) Mutation frequency was monitored by screening for resistance to L-canavanine (50 µg/mL) due to loss-of-function mutations in *CAN1* on SC medium lacking arginine. (**B**) Chromosome instability at the *MAT alpha* locus was assaying by selecting for A-like fakers using a mating assay. A-like fakers were scored based on the formation of diploids between strains from the BY4742 background (*MAT alpha, TRP1*, *lys2*) and EG103 (*MAT alpha*, *trp1*, *LYS2*) by selecting for yeast prototrophic for both tryptophan and lysine. Statistical analysis employed Student’s *t*-test with ** *p* < 0.01, * *p* < 0.05.

The ability of *B. monnieri* extracts to induce an apoptosis-like effect in yeast deleted for *RAD1* [19] as well as apoptosis in some cancer cells lines [29,30,31] suggested an impact on DNA integrity. Using the *CAN1* forward mutation assay, a significant increase in the mutation rate was observed in *rad1*∆ cells exposed to *B. monnieri* extracts. Similarly, treatment with methyl methanesulfonate (MMS) or oxaliplatin (OxPT), compounds known to induce DNA damage [8,32], resulted in an enhanced mutation frequency in the *CAN1* locus, as judged by increased numbers of canavanine resistant colonies (Figure 1A). Exposure to *B. monnieri* extracts did not promote increased chromosome instability in *rad1*∆ cells compared to WT. Although treatment with MMS or oxaliplatin produced a significant increase in the appearance of A-like fakers due to recombination at the *Mat alpha* locus (Figure 1B). These results are consistent with a connection between DNA damage but not chromosome instability and exposure to *B. monnieri* extracts. Sensitivity of *rad1*∆ cells to *B. monnieri* extracts was complemented by episomal expression of *RAD1* indicating the effect was not due to a mutation at a second site in the genome (Figure S1).

### 2.2. B. monnieri Extracts Promote Vacuole Damage

**Figure 2:**
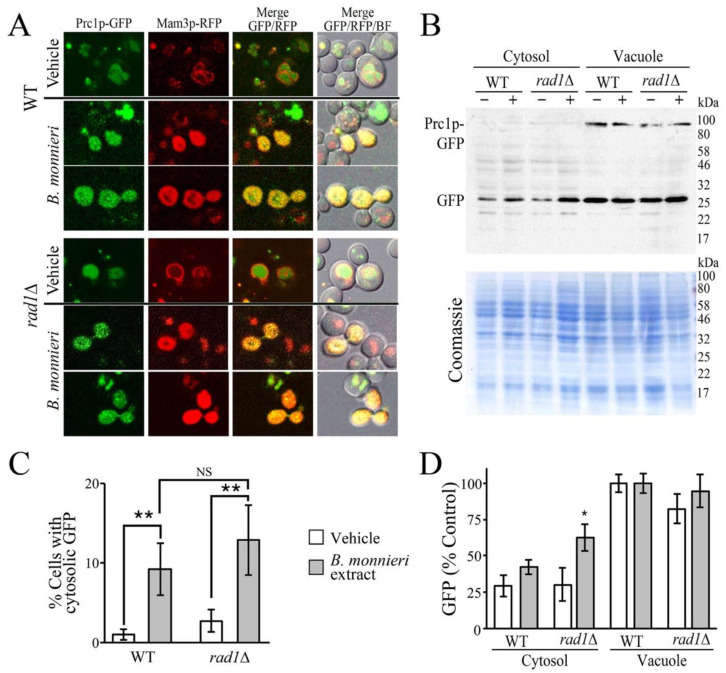
Vacuoles exhibit damage following exposure to *B. monnieri extracts.* (**A**) Strains WT (BY4742) and *rad1*∆ (12806) co-transformed with *PRC1*-GFP (pWC022) and *MAM3*-RFP (pLJ521) expression plasmids were grown in synthetic medium lacking uracil and leucine with 2% glucose in the presence of 2% DMSO and 0.2% Tween 80 (vehicle control) or 325 µg/mL *B. monnieri* extracts for 16 h prior to visualization at a magnification of 60×. (**B**) Quantitation of cells exhibiting vacuolar disruption was performed using approximately 100 cells per sample. *B. monnieri* extracts significantly increased vacuolar disruption relative to vehicle control in both WT and *rad1*∆ yeast. Values are mean ± SD (*n* = 3). (**C**) Cytoplasmic and crude vacuolar fractions were prepared from strains WT (BY4742) and *rad1*∆ (12806) transformed with *PRC1*-GFP (pWC022) grown with vehicle (−) or 325 µg/mL *B. monnieri* extract (+) for 16 h. Visualization utilized immunoblots with and anti-GFP anti-body (GFP B-2). A Coomassie stained gel of the same extracts was used as a loading control. (**D**) Quantitation of GFP levels. Values are relative to WT vacuole fraction grown with vehicle. Statistical analysis employed one-way ANOVA with post-hoc Tukey test with ** *p* < 0.01, * *p* < 0.05, NS—not significant.

The vacuole is the hydrolytic compartment of yeast and has a similar function as mammalian lysosomes in the detoxification and recycling of macromolecules [33,34]. Lysosomes can also participate in the regulation of apoptosis in mammalian cells [35,36]. Similarly, the yeast vacuole has been reported to be involved in the promotion of apoptosis-like events [37]. These processes appear to be mediated by the release of lysosomal/vacuolar hydrolases into the cytosol following membrane permeabilization [38]. *B. monnieri* extracts contain saponins such as bacosides, bacopasides, bacopasaponins [25,27,39] and these compounds have the potential to promote membrane permeabilization [40,41], which has been implicated in lysosomal mediated apoptosis [42,43]. We observed that exposure of yeast cells to *B. monnieri* extracts was found to increase the appearance in the cytoplasm of the vacuolar Prc1p-GFP fusion (Figure 2A,B), suggesting permeabilization of the vacuole membrane. The vacuolar membrane marker Mam3p-RFP was utilized to examine the structure of vacuoles in cells showing cytoplasmic Prc1p-GFP. It was observed that cells exhibiting diffuse Prc1p-GFP fluorescence had a loss of vacuolar structure. The effect of *B. monnieri* extracts was not selective to yeast deleted for *RAD1* as the WT strain also exhibited an increased number of cells with diffuse Prc1p-GFP. Although the number of cells with diffuse GFP fluorescence was greater in the *rad1*∆ samples, the difference did not exhibit statistical significance. Analysis of cytoplasmic and vacuolar fractions indicated that exposure to *B. monnieri* extracts causes an increase in release of GFP from the vacuole (Figure 2C,D). The Prc1p-GFP fusion was not observed in the cytoplasmic fraction, perhaps due to its relatively large size of 87 kDa; however, free GFP derived from this fusion protein was released into the cytoplasm. The *rad1*∆ strain exhibited a statistically significant increase in cytoplasmic GFP following exposure to *B. monnieri* extracts*,* relative to the WT strain.

### 2.3. Vacuole Permeabilization with Chloroquine Does Not Mimic Effects of B. monnieri Extracts

**Figure 3:**
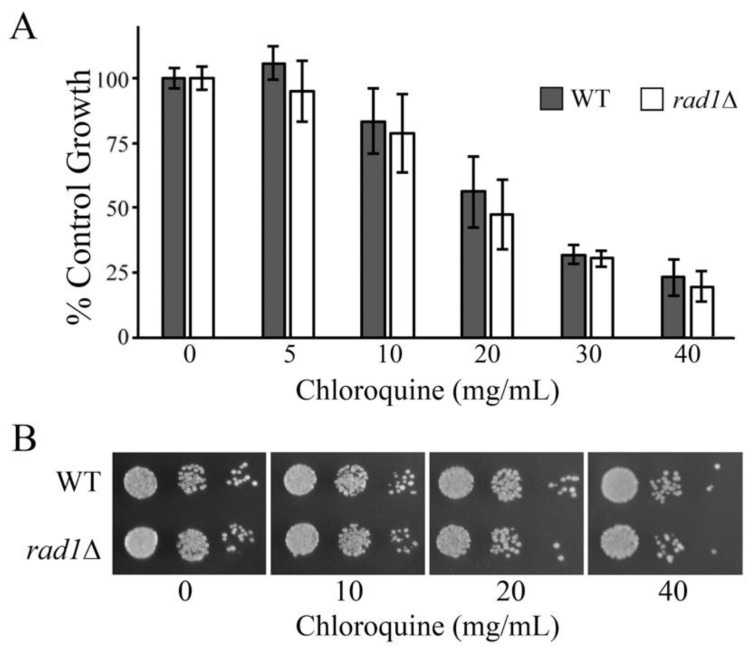
Chloroquine is not specifically toxic to yeast deleted for *RAD1*. (**A**,**B**) Yeast strains WT and *rad1*∆ (12806) were grown in synthetic medium with 2% glucose in the presence of 2% DMSO and 0.2% Tween 80 (vehicle control) or chloroquine at the concentrations listed. Values are mean ± SD (*n* = 3). Growth was monitored by measuring OD at 600 nm with vehicle control (0) set at 100%. Statistical analysis employed Student’s *t*-test and no significant differences were observed between WT and *rad1*∆ samples. (**B**) Serial dilutions of liquid cultures from were spotted onto solid synthetic medium with 2% glucose. The volume of each culture used was based on the OD 600 nm of vehicle samples for each strain background. For vehicle samples 10^5^, 10^4^, and 10^3^ cells were placed onto agar plates and the same culture volume was used for samples treated with *B. monnieri* extracts. Samples were incubated for 3 days and photographed.

The effects of *B. monnieri* extracts on vacuolar structure suggested that increased permeabilization may contribute to toxicity in *rad1*∆ yeast. Chloroquine exposure promotes lysosomal permeabilization [44] and can be used to evaluate the contribution of vacuolar permeabilation alone to sensitzation of *rad1*∆ cells. Toxicity from chloroquine was observed in both WT and *rad1*∆ yeast cells. However, both WT and *rad1*∆ strains were similarly affected by chloroquine (Figure 3). This suggests that increased vacuolar permeabilzation alone is not sufficient to sensitize *rad1*∆ cells to the effects of *B. monnieri* extracts.

### 2.4. Deletion of PEP4 Limits Toxicity of B. monnieri Extracts in rad1∆ Yeast

**Figure 4:**
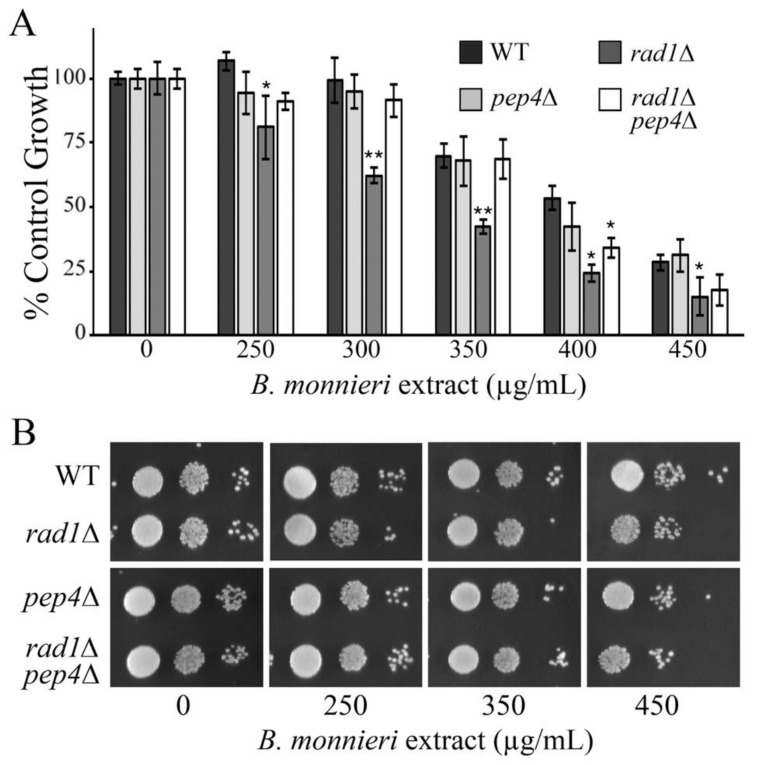
Deletion of *PEP4*, encoding a vacuolar protease, is protective against toxicity from *B. monnieri* extracts in *rad1*∆ cells. (**A**,**B**) Yeast strains WT, *rad1*∆ (12806), *pep4*∆ (12098), and *rad1*∆ *pep4*∆ (LJ467) were grown in synthetic medium with 2% glucose in the presence of 2% DMSO and 0.2% Tween 80 (vehicle control) or *B. monnieri* extracts at the concentrations listed. Values are mean ± SD (*n* = 3). Growth was monitored by measuring OD at 600 nm with vehicle control (0) set at 100%. Statistical analysis employed Student’s *t*-test with ** *p* < 0.01, * *p* < 0.05. (**B**) Serial dilutions of liquid cultures from were spotted onto solid synthetic medium with 2% glucose as described in Figure 3. Samples were incubated for 3 days and photographed.

*PEP4* encodes a vacuolar aspartyl protease (proteinase A) that is required for maturation of vacuolar proteinases [45]. If release of vacuolar proteases was involved in sensitivity of *rad1*∆ cells to *B. monnieri* extracts, then deletion of *PEP4* would be expected to have a protective role. Consistent with this idea, yeast deleted for both *RAD1* and *PEP4* show resistance to *B. monnieri* extracts to similar levels as the WT strain (Figure 4). It appears that even though vacuolar permeabilization alone (chloroquine exposure) is not sufficient to induce sensitivity of *rad1*∆, cells release of vacuolar proteases is involved.

### 2.5. Mitochondrial Structure Is Not Disrupted by Exposure to B. monnieri Extracts

**Figure 5:**
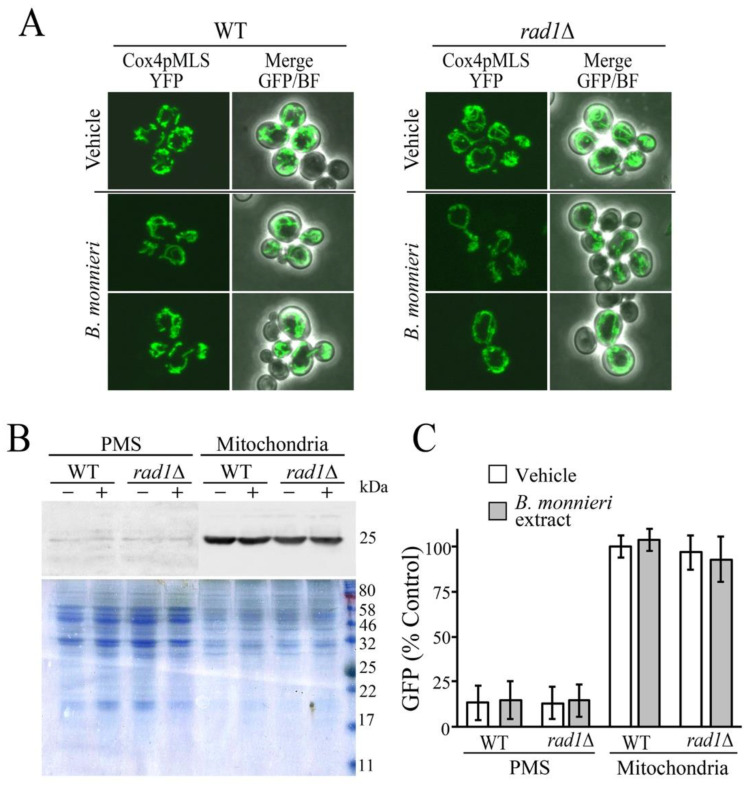
Mitochondrial morphology and integrity is not altered following exposure to *B. monnieri* extracts. (**A**) Strains WT (BY4742) and *rad1*∆ (12806) transformed with a plasmid expressing a mitochondrial matrix targeted YFP, Cox4pMLS-YFP (pLD207) were grown in synthetic medium lacking uracil with 2% glucose in the presence of 2% DMSO and 0.2% Tween 80 (vehicle control) or 325 µg/mL *B. monnieri* extracts for 16 h prior to visualization at a magnification of 60×. (**B**) Post-mitochondrial (PMS, cytosol) and mitochondrial fractions were prepared from strains as described in (**A**) treated with vehicle (−) and 325 µg/mL *B. monnieri* extract (+). Visualization utilized immunoblots with and anti-GFP anti-body (GFP B-2). A Coomassie stained gel of the same extracts was used as a loading control. (**C**) Quantitation of GFP levels. Values are relative to WT vacuole fraction grown with vehicle. Statistical analysis employed one-way ANOVA with post-hoc Tukey test, no significant differences were found between WT and *rad1*∆ samples.

The morphology of mitochondria was evaluated in WT and *rad1*∆ cells expressing YFP targeted to the mitochondrial matrix (Cox4pMLS-YFP) [46]. Mitochondria exhibited typical tubular morphology in both WT and *rad1*∆ strains grown with vehicle or *B. monnieri* extracts (Figure 5A). Analysis of YFP levels in cytoplasmic (post-mitochondrial supernatent, PMS) and mitochondrial fractions did not reveal any indication of disruption to the mitochondrial inner membrane. YFP was primarily localized to the mitochondria with only trace amounts present in the PMS fraction in all samples regardless of exposure to B. monnieri extracts (Figure 5B,C).

### 2.6. Stabilization of Plasma Membrane Does Not Prevent Toxicity from B. monnieri Extracts

**Figure 6:**
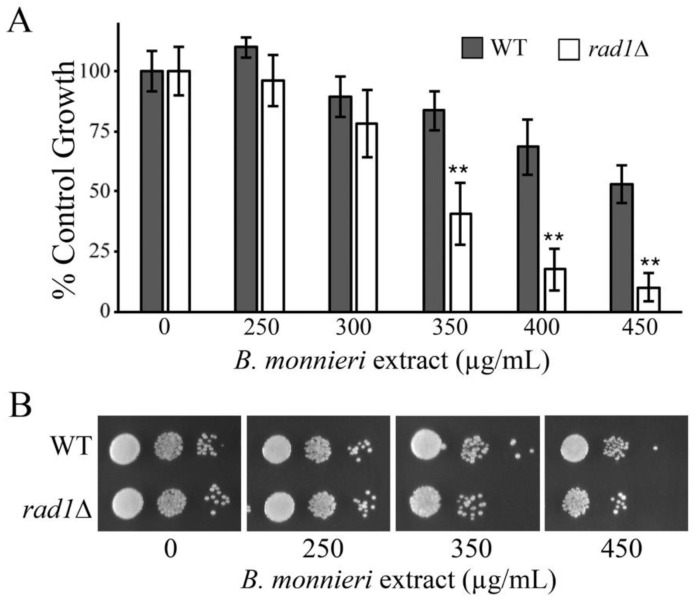
Osmotic stabilization of plasma membrane does not prevent *B. monnieri* toxicity. (**A**) Yeast strains WT and *rad1*∆ (12806) were grown in synthetic medium with 2% glucose supplemented with 0.8 M sorbitol in the presence of 2% DMSO and 0.2% Tween 80 (vehicle control) or *B. monnieri* extracts at the concentrations listed. Values are mean ± SD (*n* = 4). Growth was monitored by measuring OD at 600 nm with vehicle control (0) set at 100%. Statistical analysis employed Student’s *t*-test with ** *p* < 0.01. (**B**) Serial dilutions of liquid cultures from A were spotted onto solid synthetic medium with 2% glucose as described in Figure 3. Samples were incubated for 3 days and photographed.

Previously, we observed that *rad1*∆ cells exposed to *B. monnieri* extracts had reduced integrity of their plasma membrane [19]. To examine if the reduced plasma membrane integrity was a key element of the toxicity of *B. monnieri* extracts toward *rad1*∆ cells, sorbitol was added as an osmotic stabilizer. The growth rate of both WT and *rad1*∆ cells was reduced in medium supplemented with 0.8 M sorbitol; however, enhanced toxicity of *B. monnieri* extracts in *rad1*∆ cells was still observed (Figure 6).

### 2.7. Toxicity of B. monnieri Extracts Is Enhanced in Yeast Competent for Sterol Production

**Figure 7:**
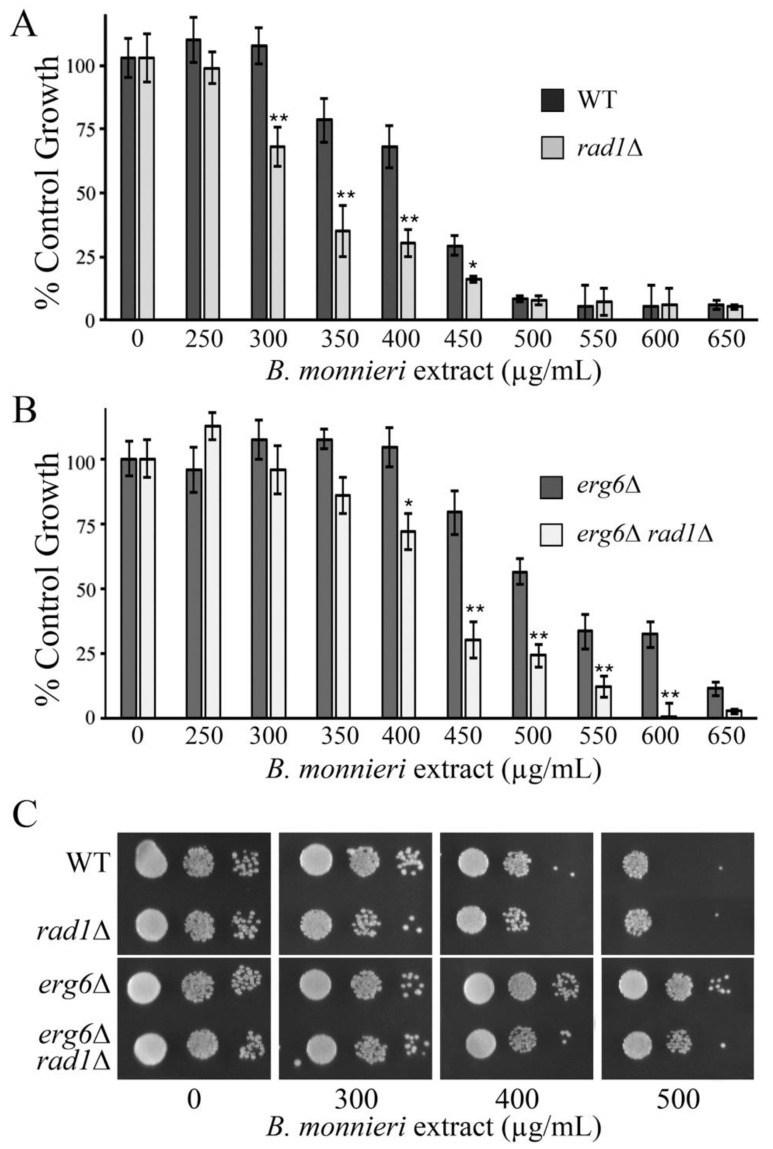
Effect of sterols on the toxicity of *B. monnieri* extracts in yeast deleted for *RAD1*. (**A**,**B**) Yeast strains WT, *rad1*∆ (12806), *erg6*∆ (PJ100), and *rad1*∆ *erg6*∆ (PJ101) were grown in synthetic medium with 2% glucose in the presence of 2% DMSO and 0.2% Tween 80 (vehicle control) or *B. monnieri* extracts at the concentrations listed. Values are mean ± SD (*n* = 3). Growth was monitored by measuring OD at 600 nm with vehicle control (0) set at 100%. Statistical analysis employed Student’s *t*-test with ** *p* < 0.01, * *p* < 0.05. (**C**) Serial dilutions of liquid cultures from A and B were spotted onto solid synthetic medium with 2% glucose as described in Figure 4. Samples were incubated for 3 days and photographed.

Several mechanisms for the toxicity of saponins, a common component of *B. monnieri* extracts*,* have been described and interactions with sterols is a common factor [47,48,49]. Yeast deleted for *ERG6* have impaired production of ergosterol, the equivalent of cholesterol in fungi. The contribution of sterols to *B. monnieri* extract toxicity was evaluated by comparing WT, *rad1*∆, *erg6*∆, and *erg6*∆ *rad1*∆ strains for sensitivity. Yeast with an intact *ERG6* gene were sensitized to *B. monnieri* extract compared to *erg6*∆ strains (Figure 7). Deletion of *RAD1* increased toxicity of *B. monnieri* extract regardless of the status of *ERG6*. It appears that an effect mediated by altered membrane composition or structure may be involved in toxicity from bioactive compounds in extracts from *B. monnieri.*

### 2.8. The Yeast Meta-Caspase Yca1p Is Required for Toxicity of B. monnieri Extracts in rad1∆ Cells

**Figure 8:**
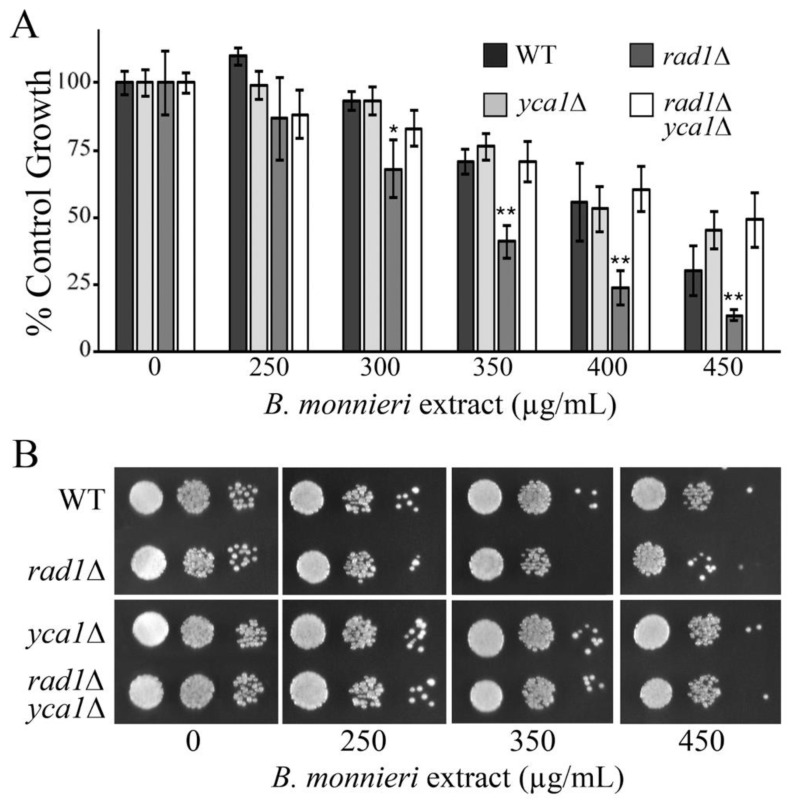
The meta-caspase Yca1p is required in *rad1*∆ cells to promote toxicity from *B. monnieri* extracts. (**A**,**B**) Yeast strains WT, *rad1*∆ (12806), *yca1*∆ (LJ464), and *rad1*∆ *yca1*∆ (LJ465) were grown in synthetic medium with 2% glucose in the presence of 2% DMSO and 0.2% Tween 80 (vehicle control) or *B. monnieri* extracts at the concentrations listed. Values are mean ± SD (*n* = 3). Growth was monitored by measuring OD at 600 nm with vehicle control (0) set at 100%. Statistical analysis employed Student’s *t*-test with ** *p* < 0.01, * *p* < 0.05. (**B**) Serial dilutions of liquid cultures from were spotted onto solid synthetic medium with 2% glucose as described in Figure 3. Samples were incubated for 3 days and photographed.

The sensitivity of *rad1*∆ cells appears to be linked to an apoptotic-like response [19]. Blocking the induction of this apoptotic-like event may be protective against toxicity from *B. monnieri* extracts. Yeast contain a metacaspase, encoded by *YCA1*, homologous to those found in mammalian cells and is involved in induction of an apoptosis-like pathway following hydrogen peroxide exposure [50]. Disruption of *YCA1* provided significant protection to *rad1*∆ cells against toxicity from *B. monnieri* extracts (Figure 8). These results confirm that *rad1*∆ yeast are sensitized to *B. monnieri* extracts due to activation of an apoptosis-like pathway.

### 2.9. Yeast Deleted for RAD52 Exhibit Synthetic Lethal Interactions with B. monnieri Extracts

**Figure 9:**
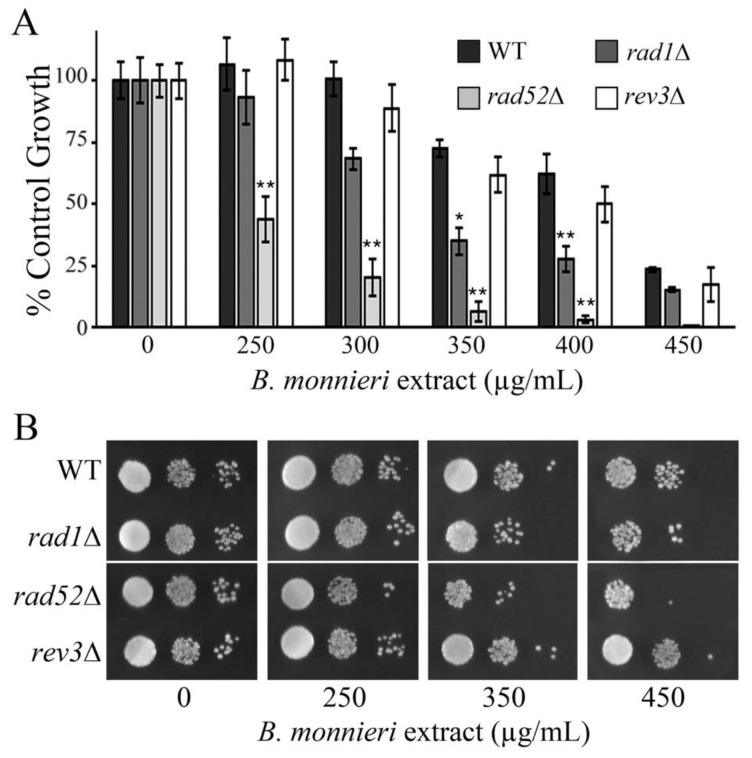
Yeast deleted for *RAD52* are sensitized to *B. monnieri extracts.* (**A**) Yeast cultures were grown as described in Figure 3 with the indicated concentration of *B. monnieri* extracts. Strains utilized include WT (BY4742), *rad1*∆ (12806), *rad52* (10540), and *rev3*∆ (12085). Values are mean ± SD (*n* = 3). Statistical analysis employed Student’s *t*-test with ** *p* < 0.01 and * *p* < 0.05. (**B**) Serial dilutions of liquid cultures from were spotted onto solid synthetic medium with 2% glucose as described in Figure 3.

A synthetic lethal interaction has been observed in *rad1*∆ yeast with *VMA6*, encoding a subunit of the vacuolar H+-ATPase (V-ATPase) important for vacuole function [51]. Interestingly, synthetic lethal interactions between *VMA6* have been reported with *RAD52* and *REV3* involved in DNA repair [52,53,54]. Rad1p and Rad52p have primary roles in double-strand break (DSB) repair [55,56] while Rev3p is involved in translesion synthesis and post-replication repair [57]. Yeast deleted for *RAD52* exhibited increased sensitivity to *B. monnieri* extracts compared to *rad1*∆ cells. In contrast, the sensitivity of *rev3*∆ cells to *B. monnieri* extracts was similar to the WT strain (Figure 9). Although a strict correlation between sensitivity to *B. monnieri* extracts and synthetic lethal interactions with *VMA6* was not observed it appears the defects in double-strand break repair may sensitize cells to the effects of compounds present in extracts from *B. monnieri.* The contribution of *VMA6* to the effects of *B. monnieri* extracts is not clear but does suggest the vacuole as a potential target.

### 2.10. Bacopasaponin C Preferentially Limits the Growth of RAD1 Deleted Yeast

**Figure 10:**
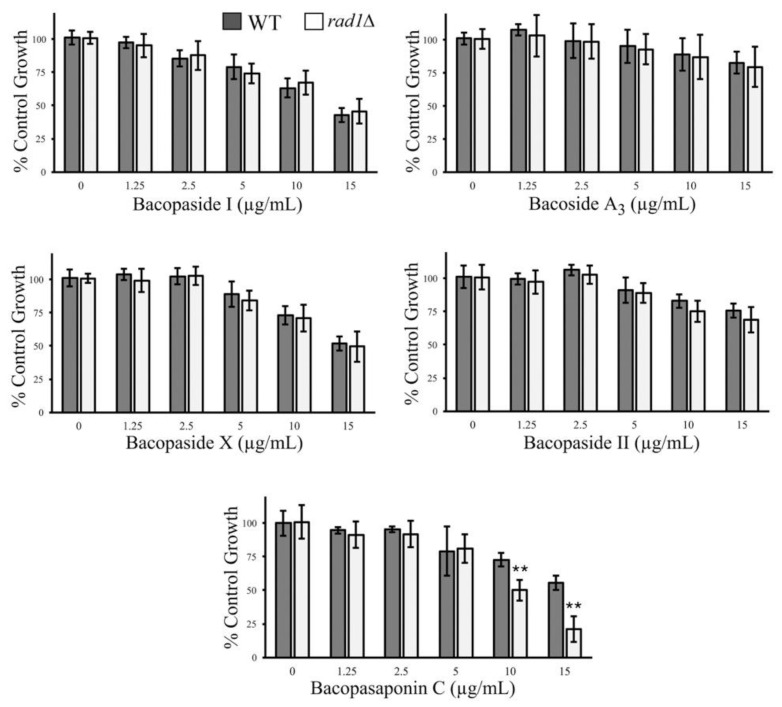
Bacopasaponin C exhibits chemical-genetic interactions with *RAD1*. Five of the major constituents of *B. monnieri* extracts were examined for synthetic lethal interactions with yeast deleted for *RAD1*. Strains utilized were WT (BY4742) and *rad1*∆ (12806). Strains were grown in synthetic medium with 2% glucose in the presence of vehicle (2% DMSO and 0.2% Tween 80), bacopaside I, bacoside A3, bacopaside X, bacopaside II, or bacopasaponin C at the indicated concentrations. Growth was monitored by measuring OD at 600 nm with vehicle control (0) set at 100%. Values are mean ± SD (*n* = 3). Statistical analysis employed Student’s *t*-test ** *p* < 0.01.

Major chemical constituents of *B. monnieri* have been previously identified including several biologically active compounds [27,58]. The most studied fraction bacoside A is a blend of bacoside A_3_, bacopasides I, II, X, and bacopasaponin C [25,39]. We examined the purified compounds from *B. monnieri,* including those found in the bacoside A fraction and bacopaside I, for chemical genetic effects with *rad1*∆ yeast. Bacoside A_3_ and bacopaside II did not cause substantial toxicity to either the WT or *rad1*∆ strain at the concentrations tested (Figure 10). Bacopaside I and bacopaside X exhibited toxicity toward yeast cells but reduced growth of both WT and *rad1*∆ cells to a similar degree. In contrast, bacopasaponin C was capable of preferentially limiting the growth of yeast deleted for *RAD1*. The growth inhibition profile of bacopasaponin C was similar to that observed for extracts from *B. monnieri*, suggesting that this compound may contribute to the synthetic lethal activity toward *rad1*∆ cells (Figure 10).

## 3. Discussion

DNA damage is a major inducer of apoptosis due to either exposure of cells to compounds that form DNA lesions or defects in DNA repair systems [59,60]. However, apoptotic pathways can be activated by several types of cellular insults [61]. Yeast, although a unicellular organism, contains genes homologous to apoptotic regulators [61,62]. This conservation of key components of apoptosis pathways in yeast has allowed its use to facilitate the understanding of basic processes that occur in mammalian cells [61].

Previously we reported that *B. monnieri* extracts induced apoptosis-like effects in yeast cells lacking the single-stranded DNA endonuclease encoded by *RAD1*. The cause for the appearance of the apoptosis-like characteristics in yeast cells deleted for *RAD1* and exposed to *B. monnieri* extracts was not identified. In this study, the potential of *B. monnieri* extracts to induce DNA mutations and chromosomal instability was evaluated using the *CAN1* forward mutation assay [63] and appearance of A-like fakers [64]. Our analysis revealed an increase in the mutation frequency following exposure of *B. monnieri* extracts*,* although chromosomal instability was not significantly increased in yeast deleted for *RAD1* compared to the WT strain. Exposure to MMS or oxaliplatin resulted in increased mutations and chromosomal instability. These findings indicated that DNA damage may be involved in the induction of an apoptosis-like events following exposure to *B. monnieri* extracts.

Several inducers have been found to promote apoptosis-like events in yeast cells [50,62,65]. In addition to the mitochondrial pathway, the yeast vacuole, similar to mammalian lysosomes, can participate in the appearance of apoptotic characteristics [37,66]. The vacuole/lysosome contains numerous hydrolases capable of degrading cellular macromolecules [34]. The release of vacuolar/lysosomal contents into the cytosol, through a process termed lysosomal membrane permeabilization (LMP), has been implicated in the induction of apoptosis in both yeast and mammalian cells [37,38]. Our analysis indicates that exposure to *B. monnieri* extracts increases the number of yeast with the diffuse appearance of a GFP fusion with Prc1p, vacuolar carboxypeptidase Y. A marker protein for the vacuolar membrane, Mam3p-RFP, was also observed with a diffuse localization in a fraction of cells exposed to *B. monnieri* extracts. It appears that treatment with *B. monnieri* extracts leads to severe vacuolar damage in some cells. This effect was observed in both wild-type and *rad1*∆ yeast cells, indicating that loss of *RAD1* is not enhancing the ability of *B. monnieri* extracts to promote vacuolar damage. Although release of GFP cleaved from the GFP-Prc1p fusion was released from *rad1*∆ cells at a higher level than WT cells. *B. monnieri* extracts do not appear to have a general effect to disrupt all intracellular organelles. Mitochondrial morphology and integrity were not altered in WT or *rad1*∆ cells following treatment with *B. monnieri* extracts. We suspect that *rad1*∆ cells are more sensitive to the effects of vacuolar damage from *B. monnieri* extracts.

One of the triggers of LMP is exposure to lysosomotropic agents that accumulate within the vacuole/lysosome and cause destabilization of the membrane [67]. Among the best-characterized compounds from *Bacopa monnieri* are triterpenoid saponins, known as bacosides [27,39]. The mechanisms for the activity of saponins typically involves the interaction with sterols, leading to membrane disruption [47,48,49]. The lipid and protein content of membranes from intracellular organelles is distinct and saponins can display specificity toward particular organelles [40]. If saponins were involved in promoting vacuolar damage, then altering the sterol content should modulate the toxicity of *B. monnieri* extracts. *ERG6*, encodes Δ(24)-sterol C-methyltransferase in the ergosterol synthesis pathway, and the *erg6*∆ strain is similar to wild-type yeast except it exhibits decreased levels of sterols [19]. Interestingly, a comparison of wild type and cells lacking *ERG6* revealed that yeast competent for sterol synthesis were sensitized to *B. monnieri* extracts relative to those with reduced sterol content. The increased sensitivity of *rad1*∆ yeast to *B. monnieri* extracts was apparent with or without an intact *ERG6* gene. Overall, these results are consistent with saponins, or other sterol interacting compounds, from *B. monnieri* promoting the synthetic lethal effects seen in the absence of *RAD1*. Chloroquine is a lysosomotropic agent and would be expected to mimic the effects of *B. monnieri* extracts if vacuolar leakage was the key event in toxicity. However, while toxic to yeast, *rad1*∆ cells are not sensitized to chloroquine relative to the wild type strain. It appears that more than vacuolar leakage is required to limit growth of cells lacking *RAD1*. Our analysis did not indicate limited permeabilization of the vacuole membrane, instead a complete loss of the vacuole structure was observed. It is possible that extensive damage to the vacuole structure is required to sensitize *rad1*∆ cells to compounds present in *B. monnieri* extracts.

Release of vacuolar components may contribute to *B. monnieri* toxicity in *rad1*∆ yeast. A major function of the yeast vacuole is to provide a secure location for digestive enzymes [34]. Release of proteases from the vacuole would be expected to lead to cell damage. *PEP4* encodes a protease important for activation of many digestive enzymes in the vacuole [45] and deletion of this gene limits toxicity of *B. monnieri* extracts on *rad1*∆ cells. The cellular insult from release of vacuolar proteases into the cytoplasm may be sufficient to induce and apoptosis-like event in yeast cells. Limiting induction of apoptosis through deletion of the gene for the yeast meta-caspase (*YCA1*) would then be expected to also be protective against toxicity of *B. monnieri* extracts. We observed a strong protective effect in *rad1*∆ cells in which *YCA1* was absent. Thus it appears that release of vacuolar proteases and induction of an apoptosis-like event are important to mediate toxicity from *B. monnieri* extracts.

To better understand why yeast deleted for *RAD1* display increased sensitivity to *B. monnieri* extracts we searched the BioGRID database [68] for synthetic lethal interactions between *RAD1* and gene deletions not involved in DNA repair. Deletion of *VMA6*, encoding a subunit of the yeast vacuolar H+-ATPase (V-ATPase) has been reported to be required for survival of *rad1*∆ yeast [69]. Interestingly, yeast deleted for *RAD52*, involved in DSB repair [53], and *REV3*, important for translesion synthesis [54], are also reported to require *VMA6* for survival [69]. Testing yeast deleted for *RAD52* or *REV3* revealed that of these strains only *rad52*∆ cells were sensitized to *B. monnieri* extract. As Rad1p is also involved in DSB repair, it is possible that deficiency in DSB repair may sensitize cells to components of *B. monnieri* extracts. Our findings are consistent with the report that yeast with impaired vacuolar function, due to deletion of V-ATPase subunits, are more prone to DNA damage compared to wild-type cells [69]. We propose that exposure to *B. monnieri* extracts*,* resulting in vacuolar damage and impaired vacuolar function, causes a synthetic sick/lethal chemical-genetic interaction in cells deficient in DSB repair, such as *rad1*∆ and *rad52*∆ yeast.

*B. monnieri* contains several bioactive natural products that may contribute to reduced proliferation of cells lacking *RAD1*. Bacosides, bacopasides, and bacosaponins have been isolated from *B. monnieri* [25,26,27] and these compounds exhibit various biological activities including neuroprotection, inhibition of water channels, and anti-tumor effects [24,70,71,72]. While our analysis of bioactive compounds from *B. monnieri* was not exhaustive, a chemical-genetic synthetic lethal effect between bacopasaponin C and *RAD1* was observed.

**Figure 11:**
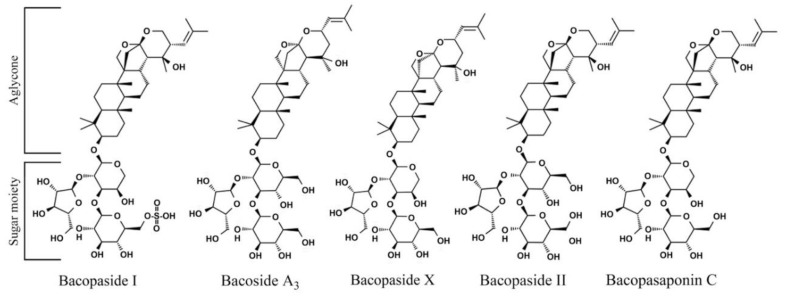
Structures of major components present in extracts from *B. monnieri.* Structures were prepared with ChemDraw Ultra, version 12.

The five bacoside compounds examined are dammarane-type triterpenoid saponins that have three sugar chains linked to a nonpolar triterpene aglycone skeleton [73]. Unique aspects of the structures of the bacopasides tested may provide a clue regarding functional groups that are involved in the chemical-genetic interactions between bacopasaponin C and *rad1*∆ yeast. The aglycone group is distinct between bacoside A_3_ and bacopaside II, with these compounds containing jujubogenin and pseudojujubogenin groups, respectively. However, bacopaside II and bacoside A_3_ do not exhibit toxicity toward yeast cells at the doses examined, indicating that the identity of the aglycone group is not sufficient to produce a toxic effect. The sugar moiety, an α-L-arabinofuranosyl-(1→2)-[β-D-glucopyranosyl-(1→3)]-α-L-arabinopyranosyl group, is shared between bacopaside X and bacopasaponin C although enhanced toxicity in *rad1*∆ yeast is only observed with bacopasaponin C. The aglycone group is different between bacopaside X and bacopasaponin C with these compounds containing jujubogenin and pseudojujubogenin, respectively (Figure 11). These findings indicate that neither the aglycone nor sugar moiety alone is sufficient to confer enhanced toxicity to *rad1*∆ yeast cells. Although the presence of pseudojujubogenin appears necessary for specificity against yeast lacking *RAD1*.

The predicted bacoside A content of *B. monnieri* extracts is approximately 6%. Bacopasaponin C comprises 0.3 to 0.6% of the material in ethanolic *B. monnieri* extracts [74]. A rough calculation would indicate that the content of bacopasaponin C from *B. monnieri* extracts utilized in our analysis at approximately 1 to 2 µg/mL. While we did not perform a quantitative analysis of bacoside content this approximate value for bacopasaonin C in the extract is 10-fold lower than that needed to produce toxicity using the purified compound. It is possible that bacopaside X, bacopaside I, or other compounds may contribute to toxicity to *rad1*∆ cells in the *B. monnieri* extract.

Overall our analysis has revealed a novel mechanism for the chemical-genetic synthetic lethal interaction between *RAD1* and bacopasaponin C present in *B. monnieri* extracts. The absence of *RAD1* in yeast results in increased mutation frequency and appears to sensitize cells to the effects of vacuolar disruption and release of proteases, such as Pep4p, following exposure to *B. monnieri* extracts. These effects together are likely sufficient to induce an apoptosis-like event that is dependent on the meta-caspase Yca1p. Among the bacoside compounds examined only bacopasaponin C preferentially limited the growth of *rad1*∆ yeast, indicating this was the active component, although other compounds present in the *B. monnieri* extract may also contribute to the toxic effect. Bacopasaponin C may have potential as a drug candidate for the treatment of colorectal cancer as well as other cancers that show impaired activity or expression of *ERCC4* (XPF), the human homologue of *RAD1*.

## 4. Materials and Methods

### 4.1. Yeast Strains and Plasmids

The majority of *S. cerevisiae* strains used in this study were derived from BY4742 (*Mat α, leu2∆0, lys2∆0, ura3∆0, his3∆1*) [75] and *KanMX4* containing single deletion strains were obtained from Open Biosystems (Layafette, CO, USA). Strain EG103 (MAT *α*, *leu2–3112*, *his3*∆*1*, *trp1–289*, *ura3–52*) has been described previously [19,76]. Gene deletions in strains LJ464 (BY4742, *yca1*∆:*URA3*), LJ465 (BY4742, *rad1*∆:*KanMX4*, *yca1*∆::*URA3*), and LJ467 (BY4742, *pep4*∆::*KanMX4*, *rad1*∆::*URA3*) were verified by in vivo PCR with a BioRad MJ Mini thermocycler (Hercules, CA, USA) using flanking primers [77]. Yeast transformations were performed using the lithium acetate procedure [78]. Cells were propagated at 30 °C either in enriched yeast extract, peptone-based medium supplemented with 2% glucose (YPD) or synthetic complete medium with 2% glucose (SC) [79]. The *CAN1* forward mutation assays utilized SC medium without arginine (SC-Arg) supplemented with 50 µg/mL canavanine (Sigma-Aldrich, St. Louis, MO, USA).

The *PRC1* coding sequence (−16 to +1596) was PCR amplified introducing a SpeI site at the 5′ end and replacing the stop codon with a NotI site. Plasmid pLJ457 containing the *PGK1* promoter and a *PHO84*-GFP fusion (*URA3*, *CEN*) [80] was digested with XbaI and NotI liberating the *PHO84* sequences. *PRC1* was ligated into the cut pLJ457 plasmid resulting in pWC022 (*PRC1*-GFP). The *RAD1* gene was PCR amplified using Pfu polymerase introducing BamHI and SalI restriction sites. The *RAD1* PCR product was digested with BamHI and SalI and ligated into pRS315 cut with the same enzymes to generate plasmid pLJ540. The *YCA1* disruption plasmid pLJ524, *PEP4* disruption plasmid pLJ538, and *RAD1* disruption plasmid pLJ539 was generated using standard techniques and utilized pRS306 (*URA3*) [81]. Transformation of yeast strains with pLJ524, pLJ538, or pLJ539 digested with BamHI resulted in the deletion of *YCA1*, *PEP4*, and *RAD1* sequences. The sequence integrity of the plasmid was verified by DNA sequencing (Macrogen, Seoul, Korea).

### 4.2. Growth Tests

Growth analysis utilized cultures in 96 well plates (50 µL each well) with shaking at 200 rpm. Cells were allowed to double at least 5 times prior to measurement of OD 600 nm. Serial dilution analysis also utilized cells grown in 96 well plates. Control cultures containing vehicle only were diluted to OD 600 nm of 1 and the same dilution was used for each treated sample in the respective set. 10-fold serial dilutions were prepared and 10 µL of each sample was spotted onto SC medium and grown for 3 days at 30 °C before being photographed. DMSO was present in all cultures at 2%, a concentration that did not inhibit the growth of yeast strains. Tween-80 was included at a concentration of 0.2% to enhance the solubility of the *B. monnieri* extract and pure compounds. Purified compounds were tested using the same procedure as for crude extracts.

### 4.3. Analysis of Mutation Rates and Chromosome Instability

Canavanine resistance and formation of A-like fakers were utilized to monitor mutation frequency and chromosome instability, respectively. Yeast were exposed to vehicle, *B. monnieri* extract at 325 µg/mL, methyl methanesulfonate (MMS) at 300 µM, and oxaliplatin (OxPT) at 2.5 mM. MMS and OxPT were used as positive controls to induce DNA damage with the selected concentrations showing approximately 50% growth inhibition in the *rad1*∆ strain. Ten independent cultures for each strain were allowed to double at least five times and the cell number was estimated using OD 600 nm.

Canavanine resistant yeast were selected by plating 500,000 cells onto SC-arginine medium supplemented with 50 mg/liter canavanine. Mutation frequency was determined using the number of canavanine resistant colonies divided by the number of total cells determined using OD 600 nm. The presence of A-like fakers was monitored using a yeast mating assay selecting for cross complementation of auxotrophic markers. The total cell number was estimated using OD 600 nm. 500,000 yeast from *erg6*∆ and *rad1*∆ *erg6*∆ in the BY4742 background were mixed with 2 million cells of the EG103 strain. A-like fakers were identified following mating by selecting for diploids (*LYS2/lys2∆, trp1∆/TRP1*) capable of growing on medium lacking both lysine and tryptophan. Results are from two independent trials and reported as colonies formed per 1 × 10^7^ cells.

### 4.4. Fluorescence Imaging

Fluorescence from the Prc1p-GFP, Mam3p-RFP, or Cox4pMLS-YFP fusions were visualized in live cells [82,83] and viewed directly at a magnification of 60× with an Olympus FV1000 or FV10-DOC confocal laser scanning microscope, equipped with universal plan super apochromat phase-contrast oil-immersion objective (Olympus Bioimaging Center, Mahidol University). To evaluate vacuolar and mitochondrial damage, 100 cells were examined for each condition. Yeast were treated with vehicle or 325 µg/mL *B. monnieri* extract (~IC_50_ concentration for *rad1*∆ yeast) for 16 h prior to visualization.

### 4.5. Cellular Fractionation

Cytosolic and vacuolar fractions were prepared following the procedure of Indge et al. [84]. Vacuolar and cytosolic fractions were separated using 10% SDS-PAGE with 20 µg of protein from each sample applied. Mitochondria and post-mitochondrial supernatents (PMS) were prepared by differential centrifugation following conversion of yeast to spheroplasts by digestion with zymolyase (20T) and lysis with a Dounce homogenizer [85]. Mitochondrial and PMS samples were separated using 15% SDS-PAGE with 15 µg of protein. Samples were transferred to nitrocellulose membranes and probed with an anti-GFP antibody, GFP-B2 (Santa Cruz Biotechnology, Inc., Dallas, TX, USA), separate gels were stained with Coomassie blue to serve as loading controls. Visualization of immunoblots used an HRP conjugated secondary antibody and enhanced chemiluminescence (ECL) detection (Merck Millipore, Burlington, MA, USA) with a G:Box Chemi XL1.4 chemiluminescence imaging system (Syngene, Frederick, MD, USA).

### 4.6. B. monnieri Extracts and Pure Compounds

*B. monnieri* powder was obtained from Herbal One Co. Ltd. (Nakhon Pathom, Thailand). *B. monnieri* (20 g) was extracted with 95% ethanol (100 mL) for 3 days. Insoluble material was removed by filtration, and the solvent was evaporated under reduced pressure using an SVC100 SpeedVac vacuum concentrator (Thermo Fisher Inc., Waltham, MA, USA). Dried extracts were weighed and stored at −20 °C prior to use. Samples were resuspended with DMSO at a concentration of 100 mg/mL. Bacoside A_3_, bacopaside I, bacopaside II, bacopaside X, and bacopasaponin C were purchased from ChromaDex Corp. (Los Angeles, CA, USA). Compounds were dissolved in DMSO at a concentration of 0.1 mg/mL.

### 4.7. Statistical Analysis

Experimental data are reported as the mean ± the standard deviation (SD). Significant differences between or among groups are indicated with, * *p* < 0.05 and ** *p* < 0.01. Data were analyzed with one-way ANOVA with post-hoc Tukey test or Student’s *t*-test as appropriate.

## Supplementary Materials

The following are available online, Figure S1: Sensitivity of *rad1*∆ cells to *B. monnieri* extracts was complemented by episomal expression of *RAD1*.

## Sample Availability

Yeast strains and plasmids are available from the authors.
