# Supplementary material for: Chemical-Genetic Interactions of Bacopa monnieri Constituents in Cells Deficient for the DNA Repair Endonuclease RAD1 Appear Linked to Vacuolar Disruption
Source: Molecules. 2021 Feb 24;26(5):1207. doi: 10.3390/molecules26051207 (PMC7956252; doi:10.3390/molecules26051207)
Supplement: Supplementary file 1 [file molecules-26-01207-s001.pdf]

Supplementary Material

Chemical-Genetic Interactions of *Bacopa monnieri* Constituents in Cells Deficient for the DNA Repair Endonuclease *RAD1* Appear Linked to Vacuolar Disruption

Chananya Huangteerakul [1], Hsu Mon Aung [1], Thitipa Thosapornvichai [1], Marisa Duangkaew [2], Amornrat Naranuntarat Jensen [3,4], Suchada Sukrong [5], Kornkanok Ingkaninan [6] and Laran T. Jensen [1,*]

[1] Department of Biochemistry, Faculty of Science, Mahidol University, Bangkok 10400, Thailand; c.huangteerakul@gmail.com (C.H.); drhsumonaung87@gmail.com (H.M.A.); pa_lim_piim@hotmail.com (T.T.)
[2] Toxicology Graduate Program, Faculty of Science, Mahidol University, Bangkok 10400, Thailand; who-miss@hotmail.com
[3] Department of Pathobiology, Faculty of Science, Mahidol University, Bangkok 10400, Thailand; amornrat.nar@mahidol.edu
[4] Center of Excellence on Environmental Health and Toxicology (EHT), Bangkok 10400, Thailand
[5] Research Unit of DNA Barcoding of Thai Medicinal Plants, Department of Pharmacognosy and Pharmaceutical Botany, Faculty of Pharmaceutical Sciences, Chulalongkorn University, Bangkok 10400, Thailand; suchada.su@chula.ac.th
[6] Department of Pharmaceutical Chemistry and Pharmacognosy, Faculty of Pharmaceutical Sciences and Center of Excellence for Innovation in Chemistry, Naresuan University, Phitsanulok 65000, Thailand; k_ingkaninan@yahoo.com
* Correspondence: laran.jen@mahidol.edu; Tel.: +66-2-201-5460

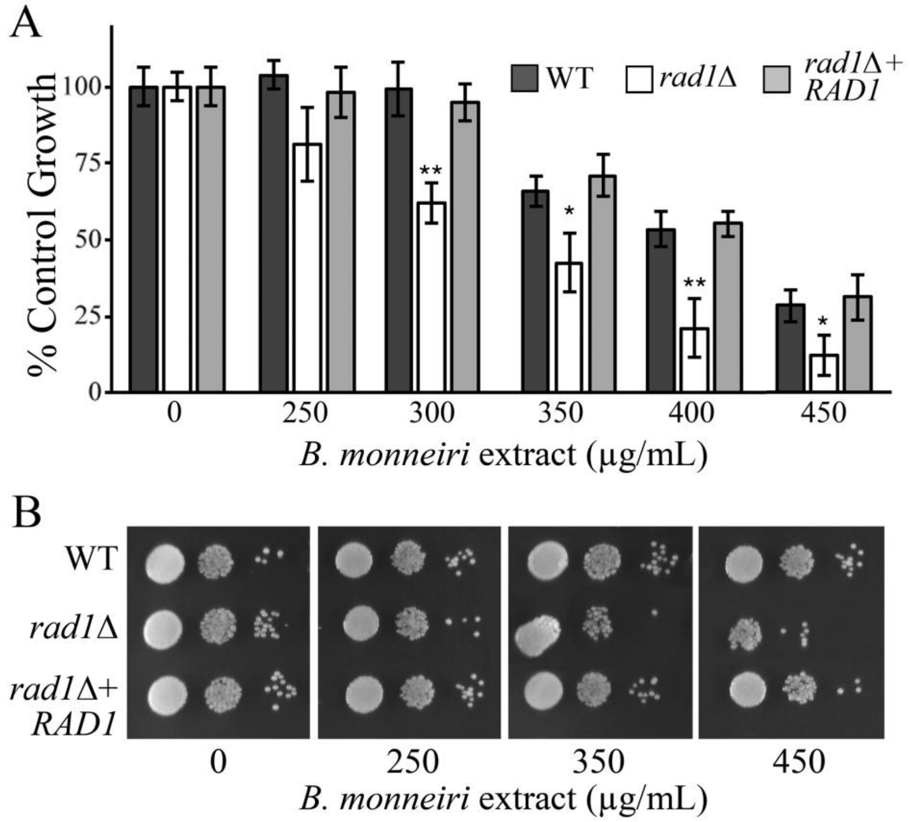

Figure S1: Sensitivity of *rad1*Δ yeast toward *B. monnieri* extracts is rescued by episomal expression of *RAD1*. (**A**,**B**) Yeast strains WT and *rad1*Δ (12806) transformed with the control plasmid (pRS315) and rad1Δ transformed with pLJ540 (*RAD1*) were grown in synthetic medium lacking leucine with 2% glucose in the presence of 2% DMSO and 0.2% Tween 80 (vehicle control) or *B. monnieri* extracts at the concentrations listed. Values are mean ± SD (n =3). Growth was monitored by measuring OD at 600 nm with vehicle control (0) set at 100%. Statistical analysis employed Student's t-test with ** $p < 0.01$, * $p < 0.05$. (**B**) Serial dilutions of liquid cultures from A were spotted onto solid synthetic medium lacking leucine with 2% glucose as described in Figure 3. Samples were incubated for 3 days and photographed.
